# e-Health in Vascular Diseases: Integrating Digital Innovation in Everyday Clinical Practice

**DOI:** 10.3390/jcm11164757

**Published:** 2022-08-15

**Authors:** Fabien Lareyre, Christian-Alexander Behrendt, Juliette Raffort

**Affiliations:** 1 Department of Vascular Surgery, Hospital of Antibes Juan-les-Pins, 06600 Antibes, France; 2 Inserm U1065, C3M, Université Côte d’Azur, 06202 Nice, France; 3 Brandenburg Medical School Theodor-Fontane, 16816 Neuruppin, Germany; 4 Research Group GermanVasc, University Medical Centre Hamburg-Eppendorf, 20246 Hamburg, Germany; 5 Department of Vascular and Endovascular Surgery, Asklepios Clinic Wandsbek, Asklepios Medical School Hamburg, 22043 Hamburg, Germany; 6 Clinical Chemistry Laboratory, University Hospital of Nice, 06001 Nice, France; 7 AI Institute 3IA Côte d’Azur, Université Côte d’Azur, 06204 Nice, France

Healthcare systems are confronted with major challenges. Demographic changes associated with advances in medicine technology have led to increased life expectancy, resulting in an increasing number of elderly people with chronic illnesses. Regional disparity with limited access to healthcare in low-density population is also a major concern for health institutions. In parallel, cultural changes with technological advancement have led to an increased demand of patients towards access care, consultations with specialists, personalized approach as well as access to medical information and communication regarding their health. 

The last decades have witnessed extensive technological progress with the development of powerful computing devices at affordable costs, the deployment and rapid spread of the Internet leading to a global connection and topped off by the use artificial intelligence (AI)-derived technology, which permits handling and analyzing large and complex data (big data) [1,2]. Digital health, also known as “e-health”, is an emerging field that has the potential to bring innovative solutions to face current challenges. It is defined as the use of information and communication technology to support the management of healthcare, which encompasses a wide range of services and systems [3,4,5]. The main domains include telemedicine/ telehealth, mobile applications (also called “m-health”), the development of smart devices such as sensors and wearables, the use of digital technology for healthcare information system or the development of integrated networks, and all are potentially enhanced using AI techniques [1,6]. Digital health represents a considerable potential for improving the management of vascular diseases and has attracted growing interest for clinicians, researchers, patients, companies, institutional and policy makers, as shown by an exponential increase in academic publications [7,8]. A substantial growth of the global market for digital health could be observed over the past decade [9]. The COVID-19 pandemic has recently added a severe strain on healthcare systems worldwide, which had to face a massive influx of patients while simultaneously adopting drastic hygiene measures and social restrictions [10]. The crisis has forced professionals and institutions to re-think their organization in order to provide appropriate and timely care and has shed light on the interest of digital health to help close the gap in health-access inequalities [11]. 

Digital tools may be useful in a wide range of settings for vascular diseases: from patient care including applications for detection, diagnosis, prognosis, treatment, follow-up or prevention to administrative tasks and the enhancement of medical information system and also to medical research and education [1,6,12,13,14,15,16]. They have the potential to improve patients’ individual health condition by improving the control of vascular diseases and associated risk factors through the enhancement of treatment compliance, adherence to behavioral changes, education for self-management and patients’ empowerment [1,6,12,13,14,15,16]. Telemedicine may facilitate access to care, reduce time and travel burden for patients and may contribute to reducing disparities in distant regions or in resource-limited areas. Finally, AI and big data analysis may help reveal new insights in the mechanisms underlying vascular diseases, build predictive models and develop precision medicine and personalized care [1,6,12,13,14,15,16]. Altogether, e-health has the potential to improve outcomes of patients with vascular diseases, with possible reduced healthcare costs at the collective level. 

While the pandemic has accelerated the use of digital technology, it has also illuminated current limitations and major challenges that remain to be faced before its implementation in daily clinical practice. This includes scientific and medical concerns as well as legal, ethical, cultural, technical and economic considerations. The main questions that people may ask first regarding digital innovation may be the following: “Is it working? Is it safe?” Generating evidence on clinical benefits, liability and safety is of utmost importance but is still challenging due to the lack of clear consensus and standardized methods as well as difficulties in organizing it at a large-scale level [1,6,12,13,14,15,16]. Digital technology raises concerns with respect to guaranteeing digital security, data protection and confidentially. While the legal framework on how to achieve these goals is still fragmented and heterogeneous, a large effort with respect to standardization and guidelines is being attempted at the European level. For instance, the European Commission most recently proposed a harmonization of rules on artificial intelligence (Artificial Intelligence Act) as well as the implementation of a new Trans-Atlantic Data Privacy Framework to allow data to flow freely and safely in the future. The implementation of digital innovation in clinical practice also requires the definition of its intended use and the determination of responsibilities, insurance coverage and re-imbursement policies, which can widely vary from one country to another. E-health is linked with technical innovation and it should be noted that it requires adequate material and infrastructures in order to provide necessary technical support and inter-operability between systems if needed. Finally, digital health uptake among patients and professionals is another cornerstone for an effective implementation of this innovation not only in everyday clinical practice but also at home. Manufacturers should carefully think and co-design their applications with all stakeholders, including health professionals, patients as well as regulatory, reimbursement and political institutions. Feedback and communication from end-users remain key points for ensuring that the applications are adapted to their needs and expectations. In terms of vascular surgery, this includes an increasing number of elderly patients and not only younger patients who are more prone to use these technologies.

Digital health is, thus, a very broad domain that is tightly linked with innovation and technology. From telemedicine to m-health, smart devices, medical information systems, integrated networks and artificial intelligence, digital health brings promising perspectives for improving the management of patients with vascular diseases. Although multiple challenges remain to be faced in order to fill the gap between technical innovation and oriented-research applications towards utilization for daily clinical practice, e-health might integrate and be part of healthcare’s digital future. 
